# Surveillance of West Nile virus infections in humans and animals in Europe, monthly report – data submitted up to 3 September 2026

**DOI:** 10.2903/j.efsa.2026.10329

**Published:** 2026-09-28

**Authors:** European Centre for Disease Prevention and Control (ECDC), European Food Safety Authority (EFSA)

**Keywords:** West Nile virus, humans, birds, equids, outbreak

## Abstract

As part of their One Health activities, the European Centre for Disease Prevention and Control (ECDC) and the European Food Safety Authority (EFSA) jointly conduct surveillance of West Nile virus (WNV) infections in humans, equids and birds and assess the epidemiological situation in Europe. This surveillance informs public health and veterinary authorities and supports timely prevention and control measures. As of 3 September 2026, intense WNV circulation has been observed in humans and animals in several European countries, although there is currently no indication of increased disease severity among human cases in EU/EEA countries. Italy remains the most affected country, reporting the highest numbers of human cases and avian outbreaks, while Greece has recorded an unusually high number of human cases for this time of year. The Netherlands has reported its first human infections since 2020, alongside outbreaks in birds and equids across a wide geographic area. In Belgium, where no human WNV infections have ever been reported, circulation in birds and equids across eight regions indicates a risk of human infection. North Macedonia is experiencing an unprecedented outbreak in the capital region, involving human cases and detections in birds. Further human cases and animal outbreaks may occur across Europe in the coming weeks. ECDC and EFSA continue to monitor the situation closely.

Suggested citation: European Centre for Disease Prevention and Control, European Food Safety Authority, 2026. Surveillance of West Nile virus infections in humans and animals in Europe, monthly report – data submitted up to 3 September 2026. Available online: https://www.ecdc.europa.eu/en/infectious-disease-topics/west-nile-virus-infection/surveillance-and-disease-data/monthly-updates

Requestor: European Commission

Question number: EFSA-Q-2025-00337

ISSN: 1831-4732

## Key findings

- The 2026 WNV season is currently seeing an intense level of circulation in both humans and animals in several European countries.
- There is currently no indication of increased disease severity in humans in EU/EEA countries.
- **Italy** remains the most affected country in 2026, reporting the most human cases and avian outbreaks. **Greece** has reported a record number of human WNV cases for this time of the year.
- **The Netherlands** reported its first human WNV infections since 2020, alongside outbreaks in birds and equids, across a broad geographic area.
- Although no human WNV cases have ever been notified in **Belgium**, ongoing circulation in birds and equids across eight regions suggests a risk for human infections.
- **North Macedonia** is experiencing an unprecedented WNV outbreak in the capital region, with human cases and detections in birds.
- Further human cases and animal outbreaks may be expected in the coming weeks in Europe.

## Epidemiological overview

### Findings from human surveillance

In 2026, and as at 3 September, countries in Europe have reported 1 086 locally acquired[1] human cases of West Nile Virus (WNV) infection. The earliest and latest dates of symptom onset among human cases were 12 May in North Macedonia and 1 September in Austria, respectively. Locally acquired cases have been reported by **Italy** (516 cases), **Greece** (284 cases, of which 11 had an unknown place of infection), **Spain** (88 cases), **Romania** (56 cases), **North Macedonia** (55 cases), **France** (36 cases), **Serbia** (18 cases), **the Netherlands** (9 cases), **Croatia** (7 cases), **Cyprus** (6 cases), **Austria** (4 cases), **Hungary** (3 cases), **Germany** (2 cases), **Albania** (1 case) and **Kosovo*** (1 case). A total of 61 deaths have been reported by **Italy** (25 deaths), **Greece** (24 deaths), **North Macedonia** (7 deaths), **Romania** (2 deaths), **Spain** (2 deaths) and **the Netherlands** (1 death).

The number of human cases reported to date (1 086 cases) exceeds the average number reported during the same period over the past decade (736 cases), indicating intense WNV circulation this year. Moreover, reporting delays may result in an under-estimation of the current burden, as the 2026 data remain provisional, whereas the 10-year average is based on consolidated data from previous years.

As at 3 September 2026, locally acquired human cases have been reported in 143 NUTS 3 regions across 15 countries, similar to the same point in 2025. However, 2024 remains the

[1] Locally acquired cases refer to cases acquired within the reporting country.
2. In this report, Europe refers to EU/EEA countries and EU-candidate countries (Albania, Bosnia and Herzegovina, Kosovo*, Montenegro, North Macedonia, Serbia and Türkiye).
*This designation is without prejudice to positions on status and is in line with UNSCR 1244/1999 and the ICJ Opinion on the Kosovo declaration of independence.

most geographically widespread WNV season on record, with 218 affected regions by the end of the season.

As in previous years, most cases were reported among males aged 65 years and above. Overall, 78% of cases were hospitalised and 60% presented with neurological symptoms, compared with 10-year averages of 87% and 65%, respectively. The case fatality rate was approximately 6%, below the 10-year average of 10%. These preliminary findings are within expected levels and do not indicate increased disease severity, although clinical outcomes are not yet available for all cases and additional deaths may be reported as the season progresses. Country-specific case fatality rates are affected by the sensitivity of the surveillance system, including screening practices of blood donors, therefore direct comparisons between countries should be made with caution.

As most WNV infections are asymptomatic or subclinical, reported case numbers represent only a fraction of the true number of infections.

## Findings from veterinary surveillance

From the veterinary perspective, 10 countries in Europe have reported 121 WNV outbreaks among equids and 280 outbreaks among birds in 2026. The earliest reported WNV outbreak among equids had a suspected start date of 30 March 2026 in France, while the most recent had a suspected start date of 29 August 2026 in Belgium. Outbreaks among equids have been reported by **France** (43 outbreaks), **Italy** (34 outbreaks), **Belgium** (14 outbreaks), **Spain** (9 outbreaks), **Greece** (8 outbreaks), **the Netherlands** (5 outbreaks), **Austria** (3 outbreaks), **Croatia** (3 outbreaks) and **Germany** (2 outbreaks). Among birds, the earliest reported WNV outbreak had a suspected start date of 31 March 2026 in Italy, while the most recent had a suspected start date of 31 August 2026 in Austria and Croatia. Outbreaks among birds have been reported by **Italy** (218 outbreaks), **Belgium** (26 outbreaks), **Croatia** (9 outbreaks), **Spain** (8 outbreaks), **Austria** (7 outbreaks), **France** (5 outbreaks), **Germany** (3 outbreaks), **North Macedonia** (3 outbreaks) and **the Netherlands** (1 outbreak). In addition, **Switzerland** submitted outbreak data to Animal Disease Information System (ADIS) for the first time in 2026, reporting one bird outbreak in Aargau (CH033).

No information was available on the equid species involved in the outbreaks reported in ADIS. The bird species most frequently associated with the reported outbreaks were the hooded crow (87 outbreaks) and the common magpie (70), followed by the carrion crow (18), the Eurasian blackbird (13), the common kestrel (10), the little owl (10), common wood-pigeon (7), the Northern goshawk (7), the yellow-legged gull (6), the Eurasian jay (5) and the European turtle-dove (5). In addition, several other bird species were associated with up to four outbreaks.

Outbreaks in equids and birds increased markedly in July and August 2026, exceeding their respective historical monthly averages (2016–2025 for equids and 2022–2025 for birds) and indicating intense WNV circulation among animals. Moreover, the outbreak numbers, particularly for August, remain provisional as reporting delays may result in additional outbreaks being notified in September or later.

As at 3 September 2026, outbreaks in birds and/or equids have been reported in 105 NUTS 3 regions across 11 countries. This exceeds the geographical spread observed at the same time in 2025, when outbreaks were reported in 88 NUTS 3 regions across 11 countries. However, the spread observed to date in 2026 remains below that observed during the corresponding period in 2024, when WNV outbreaks in birds and/or equids were reported in 131 regions across 13 countries. As noted above for human infections, 2024 also remains the most geographically widespread WNV season on record for animal outbreaks, based on the number of affected NUTS 3 regions. Of the 11 countries that submitted data to ADIS in 2026, nine had previously reported WNV outbreaks in birds and/or equids, consistent with endemic WNV circulation. **North Macedonia** and **Switzerland**, however, reported outbreaks to ADIS for the first time in 2026, with three outbreaks in birds and one in equids, respectively.

## Patterns across human and veterinary surveillance

Most affected European countries reported both human WNV infections and outbreaks in equids and/or birds. **Albania**, **Cyprus**, **Hungary**, **Kosovo***, **Romania** and **Serbia** only reported human WNV infections, while **Belgium** only reported outbreaks among equids and birds. EU candidate countries are not required to report animal outbreaks through ADIS.

In 2026, the regions listed below reported WNV activity for the first time ever. The type of first reported event is indicated in brackets for each region, distinguishing between human WNV infections, animal outbreaks or both.

**Albania** in Korcë (AL034, human, no mandatory reporting of animal outbreaks); by **Belgium** in Arr. Antwerpen (BE211, animal), Arr. de Bruxelles-Capitale/Arr. Brussel-Hoofdstad (BE100, animal), Arr. Leuven (BE242, animal), Arr. Namur (BE352, animal) and Arr. Nivelles (BE310, animal); by **France** in Aude (FRJ11, human and animal), Drôme (FRK23, human), Hauts-de-Seine (FR105, animal), Landes (FRI13, animal), Lot-et-Garonne (FRI14, animal), Pyrénées-Orientales (FRJ15, human and animal) and Seine-et-Marne (FR102, human and animal); by **Greece** in Drama (EL514, animal); by **Italy** in Trento (ITH20, human and animal) and Pescara (ITF13, human); by **Kosovo*** in Gjakovë (XK007, human, no mandatory reporting of animal outbreaks); by **the Netherlands** in Agglomeratie Leiden en Bollenstreek (NL363, human), Agglomeratie's-Gravenhage (NL361, human and animal), Delft en Westland (NL362, animal), Groot-Amsterdam (NL32B, human), Midden-Noord-Brabant (NL415, human) and Noordoost-Noord-Brabant (NL416, animal); by **North Macedonia** in Pelagoniski (MK005, human and animal) and Vardarski (MK001, human, no mandatory reporting of animal outbreaks); and **Spain** in Gran Canaria (ES705, animal).

Differences in WNV patterns across European countries may reflect a combination of ecological, climatic and surveillance-related factors. Climatic conditions, habitat characteristics and the local abundance and distribution of competent mosquito vectors and bird hosts can influence WNV circulation. Differences in surveillance design, intensity, diagnostic practices and reporting may also affect the number of human infections and animal outbreaks detected.

A selection of country-specific findings is presented below to further illustrate the heterogeneity of WNV activity across Europe.

### Italy

Italy remains the most affected country in 2026, reporting the highest number of WNV human cases (516, 48% of all reported human cases), including 25 deaths (case fatality rate: 4.8%). It also reported 34 outbreaks in equids (28% of all reported equid outbreaks) and 218 outbreaks in birds (78% of all reported avian outbreaks). Nevertheless, the number of human cases reported was lower than that reported during the same period in 2018, 2022 and 2025, based on consolidated data. The number of outbreaks in birds and equids reported to ADIS in 2026 was comparable to that reported during the same period in 2025, slightly lower than in 2024, and higher than in 2018 and 2022. Overall, 67 of Italy's 107 NUTS 3 regions are currently affected, with WNV human cases and/or animal outbreaks reported.

### Greece

Greece has reported its highest number of human WNV cases for this time of year, with 284 cases, including 24 deaths (case fatality rate: 8.5%). WNV transmission is currently most intense in the Attica NUTS 2 region, which accounts for 162 cases, with the highest numbers reported in Anatoliki Attiki (East Attica, 86 cases) and Voreios Tomeas Athinon (North Athens, 48 cases). Despite accounting for 26% of all reported human cases in Europe, Greece has reported only eight outbreaks in equids, representing 5.5 % of all reported equid outbreaks, and no outbreaks in birds.

**The Netherlands**

Nine human WNV infections have been reported in the Netherlands, including one death. These are the first human cases reported in the Netherlands since 2020, when the country documented its first-ever locally acquired human WNV infections, with seven cases reported in Utrecht (previously NL310) and one in the Arnhem/Nijmegen region (NL226). In addition, five outbreaks in equids and one in birds were reported in 2026. WNV was first reported in equids in the Netherlands in 2025. Overall, WNV infections in humans and/or animals were detected across eight NUTS 3 regions this year.

**Belgium**

WNV was first detected in Belgium in 2025 through avian surveillance, having no previous detections reported in humans or animals. That year, seven bird outbreaks were reported in Arr. Mechelen (BE212), Arr. Dendermonde (BE232), and Arr. Halle-Vilvoorde (BE241). In 2026, WNV was again detected in birds in these three regions and in five previously unaffected regions. To date, Belgium has not reported any human WNV infections. However, the ongoing circulation of the virus in animals, with 14 outbreaks among equids and 26 outbreaks among birds reported across eight NUTS 3 regions, is indicative that there is a risk for human infections to occur.

**North Macedonia**

North Macedonia is reporting an unprecedented number of human WNV infections, with 55 cases and seven deaths (case fatality rate: 13%) reported from three NUTS3 region. Two of these regions have also reported outbreaks among birds. The Skopski region, which includes the capital city of Skopje, is by far the most affected, accounting for 53 human WNV infections and three bird outbreaks. In 2026, North Macedonia reported WNV outbreak data to ADIS for the first time, with three bird outbreaks across two regions. In the past decade, the country had reported a maximum of five human WNV infections per year.

## Seasonal outlook

Given the favourable environmental conditions for WNV transmission in Europe, ECDC and EFSA expect further human cases and outbreaks in equids and birds to be reported in the coming weeks. In previous years, transmission has typically peaked in August and early September.

ECDC and EFSA continue to closely monitor the situation in Europe.

## Background

In the context of their 'One Health' activities, the European Centre for Disease Prevention and Control (ECDC) and the European Food Safety Authority (EFSA) conduct joint surveillance of West Nile virus (WNV) infections in humans, equids, and birds, while also assessing the epidemiological situation in Europe. This initiative aims to inform public health and veterinary authorities in Europe, facilitating the implementation of prevention and control measures. These measures include the deferral or testing of blood donors who have potentially been exposed to the virus, in accordance with Commission Directives Commission Directives 2004/33/EC and 2014/110/EU.

The tables and maps in this monthly report show the countries and areas where human cases of WNV infection and outbreaks among equids and birds have been reported to the European surveillance portal for infectious diseases (EpiPulse Cases) and the Animal Disease Information System (ADIS). In addition to this monthly report, ECDC provides a weekly report presenting the locally acquired WNV infections among humans in Europe[2]. Human surveillance covers EU/EEA countries as well as reporting by EU candidate and potential candidate countries.

For the analysis of ADIS outbreak data, this report includes only animal outbreaks with a 'suspicion/start date' in 2026. For analysis of historical data, when the 'suspicion/start date' was not reported, the 'confirmation date' was used instead. Outbreaks of WNV in animals shall be notified to ADIS in accordance with the provisions and definitions laid down in Commission Implementing Regulation (EU) 2020/2002. Under this Regulation, the reporting obligation applies to EU Member States; however, data from non-EU countries may also be available in ADIS. As human surveillance does not cover Switzerland, animal outbreaks reported to ADIS by this country are presented exclusively in the 'Findings from veterinary surveillance' section. Swiss data are not included in the maps, table, or figures in the current report. A notified outbreak may include one or more WNV cases. Since there is no specific case definition for WNV infection in animals, the general definition provided in Article 9 of Commission Delegated Regulation (EU) 2020/689 applies. When comparing the number of WNV outbreaks in birds reported in 2026 with historical data, a four-year average (2022–2025) was used – rather than the 10-year average applied to equids – since mandatory reporting of WNV outbreaks in birds only began in April 2021.

Surveillance of WNV in mosquitoes can support the detection of local virus circulation and complement human and animal surveillance findings. However, because these data are not systematically collected and reported at European level, they are not included in this report.

The full automated report, including maps, figures and a table, is available online at the following link:

https://www.ecdc.europa.eu/en/infectious-disease-topics/west-nile-virus-infection/surveillance-and-disease-data/monthly-updates

**EFSA staff consulted (in alphabetical order)**: Giusi Amore, Inmaculada Aznar Asenzio, Frank Verdonck
